# Workplace neighbourhood built environment and workers’ physically-active and sedentary behaviour: a systematic review of observational studies

**DOI:** 10.1186/s12966-020-01055-x

**Published:** 2020-11-20

**Authors:** Chien-Yu Lin, Mohammad Javad Koohsari, Yung Liao, Kaori Ishii, Ai Shibata, Tomoki Nakaya, Gavin R. McCormack, Nyssa Hadgraft, Neville Owen, Koichiro Oka

**Affiliations:** 1 Graduate School of Sport Sciences, Waseda University, 2-579-15 Mikajima, Tokorozawa, Saitama 359-1192 Japan; 2 Faculty of Sport Sciences, Waseda University, Tokorozawa, Japan; 3 Melbourne School of Population and Global Health, The University of Melbourne, Melbourne, Australia; 4 Behavioural Epidemiology Laboratory, Baker Heart & Diabetes Institute, Melbourne, Australia; 5 Department of Health Promotion and Health Education, National Taiwan Normal University, Taipei, Taiwan; 6 Faculty of Health and Sport Sciences, University of Tsukuba, Tsukuba, Japan; 7 Graduate School of Environmental Studies, Tohoku University, Sendai, Japan; 8 Department of Community Health Sciences, Cumming School of Medicine, University of Calgary, Calgary, Canada; 9 Centre for Urban Transitions, Swinburne University of Technology, Melbourne, Australia

**Keywords:** Worksite, Employee, Walkability, Physical activity, Sitting

## Abstract

**Background:**

Many desk-based workers can spend more than half of their working hours sitting, with low levels of physical activity. Workplace neighbourhood built environment may influence workers’ physical activities and sedentary behaviours on workdays. We reviewed and synthesised evidence from observational studies on associations of workplace neighbourhood attributes with domain-specific physical activity and sedentary behaviour and suggested research priorities for improving the quality of future relevant studies.

**Methods:**

Published studies were obtained from nine databases (PubMed, Web of Science, PsycINFO, Scopus, Transport Research International Documentation, MEDLINE, Cochrane, Embase, and CINAHL) and crosschecked by Google Scholar. Observational studies with quantitative analyses estimating associations between workplace neighbourhood built environment attributes and workers’ physical activity or sedentary behaviour were included. Studies were restricted to those published in English language peer-reviewed journals from 2000 to 2019.

**Results:**

A total of 55 studies and 455 instances of estimated associations were included. Most instances of potential associations of workplace neighbourhood built environment attributes with total or domain-specific (occupational, transport, and recreational) physical activity were non-significant. However, destination-related attributes (i.e., longer distances from workplace to home and access to car parking) were positively associated with transport-related sedentary behaviour (i.e., car driving).

**Conclusions:**

The findings reinforce the case for urban design policies on designing mixed-use neighbourhoods where there are opportunities to live closer to workplaces and have access to a higher density of shops, services, and recreational facilities. Studies strengthening correspondence between the neighbourhood built environment attributes and behaviours are needed to identify and clarify potential relationships.

**Protocol registration:**

The protocol of this systematic review was registered on the International Prospective Register of Systematic Reviews (PROSPERO) on 2 December 2019 (registration number: CRD42019137341).

**Supplementary Information:**

The online version contains supplementary material available at 10.1186/s12966-020-01055-x.

## Background

Many desk-based workers spend the majority of their working hours being sedentary; this is markedly greater than the proportion of time spent sedentary during non-working hours [1, 2]. An ecological model of health behaviour suggests that workers’ physically-active and sedentary behaviours are influenced by multiple factors [3]. Among the multiple influences, built environment factors can be particularly influential on desk-based workers’ sedentary behaviour [4]. The workplace built environment comprises the built environment attributes inside (e.g., workstations and spatial layout of buildings) and on the land parcel of the workplace building (e.g., workplace-exclusive car parking) and the neighbourhood surrounding the workplace (e.g., neighbourhood walkability and destination access). Previous reviews relating to the workplace built environment have mainly focused on examining inside environments as potential influences on physically-active and sedentary behaviours during work time [4–7]. However, workplace neighbourhood environment may also be an important influence on the physical activity and sedentary behaviours of workers. Given there is greater capacity for workers to undertake moderate-to vigorous-intensity physical activity (e.g., walking and cycling) in the neighbourhood around and beyond the immediate workplace setting [8], the influence of workplace neighbourhood environment merits examination [9].

Synthesising the broader body of empirical evidence relating to workplace neighbourhood built environment is essential for informing urban design policies to support physical activity and reduce sedentary behaviours among workers. A previous systematic review examining associations of workplace built environments, both inside and neighbourhood attributes, with physical activity and sedentary behaviour reported inconclusive findings for neighbourhood attributes [10]. However, the search strategy used did not employ specific terms relating to characteristics of the neighbourhood built environment such as neighbourhood walkability, destinations, and safety. Furthermore, the previous findings did not distinguish the purpose of active and sedentary behaviours (e.g., for occupation, transport, or recreation). Therefore, the associations may be confounded as the environmental correlates varied by domains [11, 12].

Therefore, the aim of our review was to examine findings from observational studies and synthesise current evidence on associations of workplace neighbourhood built environment attributes (including those not located on the workplace precinct), with domain-specific physical activity and sedentary behaviour among desk-based workers. We further provided suggestions based on the results for improving the evidence on urban design policies to influence workers’ physical activity and sedentary behaviours.

## Methods

The protocol of this systematic review was published on the International Prospective Register of Systematic Reviews (PROSPERO) on 2 December 2019 (registration number: CRD42019137341).

### Database search strategy

This systematic review was conducted in October 2019 following the Preferred Reporting Items for Systematic Reviews and Meta-Analyses (PRISMA) guidelines [13]. Systematic searches were conducted in nine databases: PubMed, Web of Science, PsycINFO, Scopus, Transport Research International Documentation, MEDLINE, Cochrane, Embase, and CINAHL. We also used Google Scholar to confirm missing studies. The last search was carried out in the beginning of January 2020. Three sets of search terms were used: environment variables (e.g., workplace, worksite, and neighbourhood), physical activity (physical activity and walking), and sedentary behaviour (sedentary behaviour and prolonged sitting). Supplementary Material 1 shows all the search terms and syntax used for the search.

### Screening

**Fig. 1:**
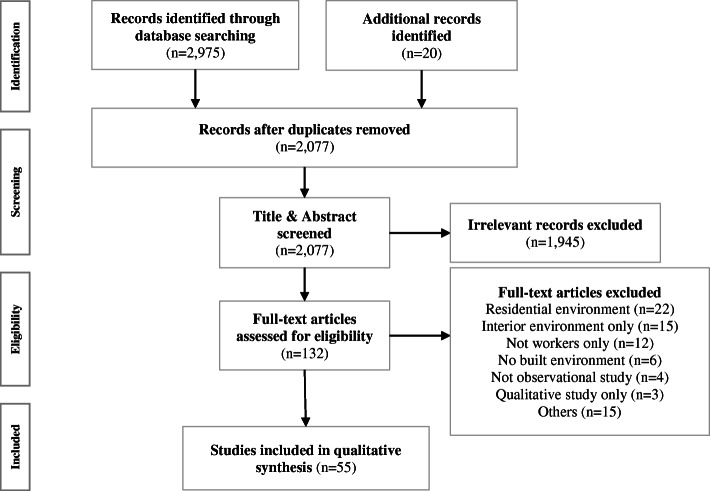
Flow chart of the literature search process

The database search produced 2077 articles after removing duplicates. They were screened by two independent reviewers (CYL and YL). The inclusion criteria were: i) published after 2000 in peer-reviewed journals; ii) full-text was written in English; iii) observational studies with quantitative analyses, and iv) estimated associations of self-reported or objective built environmental measures around the workplace with physical activity or sedentary behaviour among workers. We conducted the review on articles published after the year 2000 because studies on this topic began to emerge around that time [10]. Those studies which examined only other environmental measures, for example, the social (e.g., organisational support), informational (e.g., posters or programs), and interior (e.g., workstations) environments, which were not incorporated with neighbourhood workplace built environment were excluded. We targeted workers who mostly conduct sedentary desk-based work in a primary work location (e.g., office workers), so studies that focused on factory workers, drivers, and clinical nurses were excluded. The screening process based on title and abstract removed 1945 articles. Two independent reviewers (CYL and YL) read the full text of the remaining 132 articles to check their eligibility. This process identified 55 articles to be included in the review by the two reviewers [14–68]. The consistency of the screening process between the two reviewers was over 95%. Any uncertainty of inclusion of articles was discussed with a third reviewer (MJK) until consensus was reached. Figure 1 shows the flow chart illustrating the process of database search and screening.

### Data extraction

All relevant information from the full-texts of the eligible articles was extracted by one reviewer (CYL) and cross-checked by the second reviewer (YL). We extracted the following information: study location; sample; study design; built environment attributes and measurement methods (i.e., perceived or objectively-measured); outcome variables and measurement methods (i.e., reported or objectively-measured); covariates; and results. Associations between built environment attributes and outcome variables were reported in various ways, including Spearman or Pearson correlation coefficients, regression beta coefficients, and odds ratios.

The outcome variables were categorised into total and different domains of physical activity and sedentary behaviour, including occupational; transport; and recreational physical activity or sedentary behaviour, based on the definitions employed in each study.

**Fig. 2:**
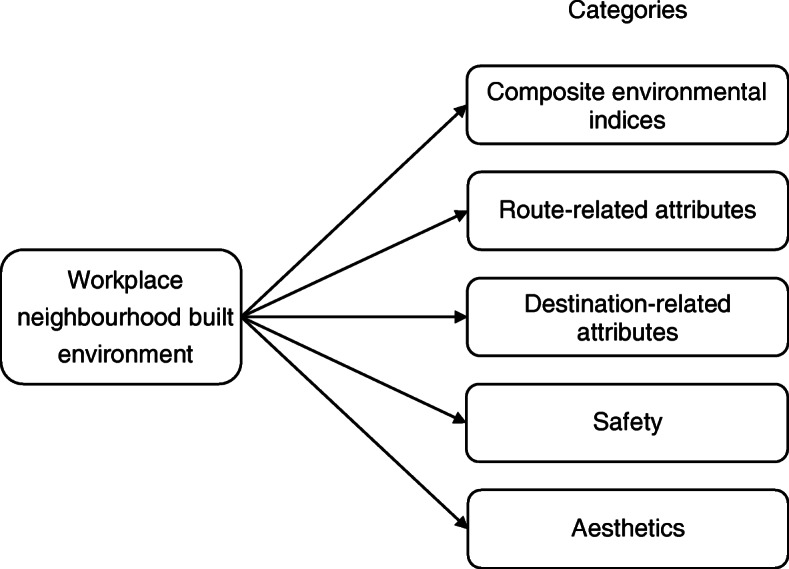
The framework applied in this systematic review

Adapting the categories of neighbourhood built environment attributes employed in the previous reviews [11, 12, 69], we divided the workplace neighbourhood built environment into five categories (Fig. 2):

i) Composite environmental indices: a) a composite index including multiple neighbourhood built environments across different types (e.g., walkability calculating the density, land use mix, and connectivity); and, b) a composite index mixing neighbourhood built environment with other attributes such as interior built environment and/or workplace policies together (e.g., a scale measuring facilities around workplace, social climate, and organisational supports).
ii) Route-related attributes: these included routes for pedestrians or cyclists and street connectivity or intersection density.
iii) Destination-related attributes: these included the presence, density, and diversity of destinations which were assumed to increase physical activity such as shops, transport stops, recreational facilities; and the distance between workplace and home or city centre. Of note, the presence of car parking, which was specifically examined as a matter of convenience to drive cars (i.e., a type of sedentary transport behaviour), was also examined in some studies.
iv) Safety: these comprised a low volume of traffic for pedestrians and bicyclists, low crime rates, and lighting along the commuting routes.
v) Aesthetics: these included general aesthetics, greenness, and being free of litter.

### Quality assessment

The scientific rigour of the selected articles was reviewed and assessed by two independent reviewers (CYL and YL), using the study quality assessment tool for observational cohort and cross-sectional studies [70]. The details of this assessment tool are shown in Supplementary Material 2. Each article was assessed against 14 criteria, including research aim, participants, measurements, and statistical analysis. Based on the guidance for the assessment tool [70], the research aim was assessed based on its importance and explicit description. Detailed information such as the demographic, location, and inclusion criteria provided showing a higher probability to replicate the study. Studies using a reliable and valid measurement of variables, employing multiple measurements, and considering the potential covariates typically receive higher quality scores. Each article was given a quality rating of good, fair, or poor according to the scoring guidelines. Disagreements were discussed between the two raters until consensus was reached. Most of the reviewed studies were of good (58.2%) or fair (40.0%) quality; therefore, we did not weight study findings based on their rigour. There was moderate agreement between the two independent raters on the quality assessment of the included studies; the percentage of overall agreement was 85.5%, and Cohen’s Kappa coefficient was 0.69.

### Synthesis of research findings

The associations of workplace built environment attributes with physical activity, or sedentary behaviour were coded into “+” (significant positive association), “−” (significant negative association), and “N” (non-significant association). If a study reported findings from several models, only the results of the most adjusted models were used. If a study reported findings from a composite score as well as its components, the results of each component were extracted. Furthermore, if a study showed results for both the overall sample and subsamples, the results from the subsamples were primarily extracted. We summarised the findings for each domain of physical activity and sedentary behaviour separately. This review considered an association to be significant if the *p*-value of an observed effect was < 0.05.

## Results

### Study characteristics

Cross-sectional studies accounted for 85.5% of the 55 included articles (Table 1). The period of follow-up implemented in the eight longitudinal studies ranged from 1 month [20, 67, 68] to 7 years [59]. Around half of the studies reviewed (*n* = 26) were included in the recent review by Zhu et al. [10], with the other half of the studies (*n* = 29) being novel to our review. Most of the studies were undertaken in the USA (*n* = 25, 45.5%) and the UK (*n* = 13, 23.6%), accounting for around 70% of all studies.

**Table 1:** Characteristics and findings of observational studies (*n* = 55) examining associations of workplace neighbourhood built environment attributes with physical activity/sedentary behaviour

The lead author (Year)	Sample	Sample and Study design	Built environment attributes	PA and SB	Results of the most adjusted models	Covariates
Adams (2016) [49]	676 employed adults, UK	Recruited employees through five employers in England; Cross-sectional	i. Walking routes (Route-related; P) ii. Walking pavements (Route-related; P) iii. Maintained pavements (Route-related; P) iv. Safe to cross the road (Safety; P) v. Dangerous traffic for walking (Safety; P) vi. Crime rate (Safety; P) vii. Routes are well lit (Safety; P) viii. Free of litter/graffiti (Aesthetics; P) ix. Walking routes are well signposted (Safety; P) x. Public transport (Destination-related; P)	a. Time spent walking to and from work (Transport PA; R)	i-a. + ii-a. + iii-a. + iv-a. N v-a. N vi-a. N vii-a. N viii-a. N ix-a. N x-a. +	Sex, age, car ownership, distance lived from work, free car parking at work, and organisation
Adams (2017) [55]	1544 employed adults, UK	Recruited employees through five employers in England; Cross-sectional	i. Distance to home (Destination-related; P)	a. Time spent walking to and from work (Transport PA; R)	i-a. N	Age, car ownership, free car parking at work, work-related PA, occupation, work pattern, perceived barriers, and psychosocial factors
Adlakha (2015) [44]	2015 employed adults, USA	A multistage sampling frame was used to randomly select adults form list-assisted telephone random-digit-dialing methods; Cross-sectional	i. Healthy restaurants (Destination-related; P) ii. Transit stop (Destination-related; P) iii. Sidewalks (Route-related; P) iv. Shops, stores, or markets (Destination-related; P) v. Facilities to bicycle (Route-related; P) vi. Recreation facilities (Destination-related; P) vii. Crime rate (Safety; P) viii. Dangerous traffic for pedestrian (Safety; P)	a. Work PA (Occupational PA; R) b. Travel PA (Transport PA; R) c. Leisure PA (Recreational PA; R) d. Total PA (Total PA; R)	i-a. N; i-b. +; i-c. +; i-d. N ii-a. N; ii-b. N; ii-c. N; ii-d. + iii-a. N; iii-b. N; iii-c. N; iii-d. + iv-a. N; iv-b. +; iv-c. N; iv-d. N v-a. +; v-b. +; v-c. +; v-d. + vi-a. N; vi-b. +; vi-c. +; vi-d. N vii-a. –; vii-b. N; vii-c. N; vii-d. N viii-a. N; viii-b. N; viii-c. N; viii-d. N	Sex, age, ethnicity, education, and income
Almeida (2014) [37]	6261 employed adults, USA	Recruited employees in working in medium-sized workplaces in Virginia and Colorado; Cross-sectional	i. Outdoor space (Composite index; O)	a. Moderate activity and strength-training activities (Total PA; R)	i-a. N	Sex, age, ethnicity, and education
Badland (2008) [18]	364 employed adults not working from home, New Zealand	A random sample selected from electric telephone white pages; Cross-sectional	i. Residential density (Destination-related; O) ii. Mixed land use (Destination-related; O) iii. Street connectivity (Route-related; O) iv. Distance to home (Destination-related; O)	a. Transport-related PA (Transport PA; R)	i-a. N ii-a. N iii-a. + iv-a. –	Sex, age, ethnicity, education, household income, and require automobile for work
Badland (2010) [22]	1188 employed adults not working from home, New Zealand	A random sample selected from electric telephone white pages; Cross-sectional	i. Car parking (Destination-related; P) ii. Workplace located in an urban area (Destination-related; O) iii. Distance to home (Destination-related; O)	a. Commuting to work by public transport (Transport PA; R)	i-a. – ii-a. + iii-a. N	Sex, age, sample weighting, residential accessibility to public transport, access to private automobile, current driving license, and require automobile for work
Barrington (2015) [45]	1007 employed adults, USA	Recruited employees working in the Seattle area through workplaces; Longitudinal (follow-up: 2 years)	i. Intersections (Route-related; O) ii. Residential units (Destination-related; O) iii. Food destinations (Destination-related; O) iv. Activity destinations (Destination-related; O)	a. Total free-time PA (Recreational PA; R) b. Total walking (Total PA; R)	i-a. N; i-b. N ii-a. N; ii-b. + iii-a. NR; iii-b. N iv-a. N; iv-b. N	Sex, age, ethnicity, education, household income, manual occupation, intervention arm, worksite parcel size, and worksite internal environment variables, and worksite SES
Batista Ferrer (2018) [60]	654 employed adults, UK	A convenience sample of employees was recruited from 87 workplaces in urban areas in England and Wales; Cross-sectional	i. Distance to home (Destination-related; O) ii. Perceptions of the commuting environment (Composite index; P)	a. Incorporating PA during the commute (Transport PA; O) b. Commuting to work by walking (Transport PA; O) c. Commuting to work by public transport (Transport PA; O)	i-a. –; i-b. –; i-c. NR ii-a. NR; ii-b. NR; ii-c. +	BMI and occupational activity (for a) Workplace, access to car, and availability of workplace car parking (for b) Age, access to car, workplace, availability of workplace car parking, and combines commute with caring responsibilities (for c)
Biswas (2018) [61]	60,650 employed adults, Canada	A multistage sampling frame was used to select households across Canada randomly; Cross-sectional	i. Combination of all (Composite index; P) ii. Combination of walking and playing place (Composite index; P) iii. Combination of walking place, gym, fitness class, showers/change rooms, and health programs (Composite index; P) iv. Combination of walking place, showers/change rooms, and health programs (Composite index; P) v. Combination of walking place and showers/change rooms (Composite index; P)	a. Leisure-time PA (Recreational PA; R)	i-a. + ii-a. + iii-a. + iv-a. + v-a. N	Sex, age, ethnicity, marital status, immigrant, education, BMI, dietary intake, smoker status, alcohol consumption, perceived health and mental health, income, hours worked per week, working at home, job stress, physical demands of work, and season
Bjorkelund (2016) [50]	709 employed parents not working from home, Norway	Recruited employed parents of children in 6th and 7th graders at 27 randomly selected schools in two Norwegian counties; Cross-sectional	i. Distance to home (Destination-related; P) ii. Traffic safety (Safety; P)	a. Walking to work (Transport PA; R) b. Cycling to work (Transport PA; R) c. Driving to work (Transport SB; R)	i-a. –; i-b. –; i-c. + ii-a. +; ii-b. +; ii-c. N	Sex, education, ethnicity, access to cars/bikes, and attitudes (for a and b) Sex, education, ethnicity, access to cars/bikes, attitudes, and leisure-time PA (for c)
Bopp (2012) [29]	375 employed adults, USA	Recruited local employees working in Manhattan, Kansas, through community listservs, links from local websites, and fliers; Cross-sectional	i. Travel time to home (Destination-related; P) ii. Lack of sidewalks (Route-related; P) iii. Difficult terrain (Safety; P)	a. Walking to work (Transport PA; R) b. Biking to work (Transport PA; R) c. Driving to work (Transport SB; R)	i-a. –; i-b. N; i-c. + ii-a. N; ii-b. N; ii-c. N iii-a. N; iii-b. N; iii-c. N	Sex, age, ethnicity, education, self-efficacy, ecological friendly attitude, employment level, occupation classification, employment length, perceptions of co-worker’s active, barriers, and motivations
Bopp (2013) [31]	1234 employed adults not working from home, USA	A convenience sample was recruited in medium-large cities in the mid-Atlantic region of the U.S. through email addresses directly or listserv, e-newsletter, or mass email; Cross-sectional	i. Lack of bike lanes (Route-related; P) ii. Lack of walking/biking paths (Route-related; P) iii. Lack of sidewalks (Route-related; P) iv. Traffic volume (Safety; P) v. Crime level (Safety; P) vi. Difficult terrain (Safety; P) vii. Distance to home (Destination-related; P)	a. Work-related active commuting (Transport PA; R)	i-a. N ii-a. N iii-a. N iv-a. N v-a. N vi-a. N vii-a. N	Sex, age, ethnicity, marital status, BMI, number of children, number of chronic disease, active commuting beliefs, perceived behavioral control, self-efficacy, income, employment categories, employment length, number of cars in the household, social support, and residential environments
Bopp (2014) [38]	709 employed women not working from home, USA	A convenience sample was recruited in medium-large cities in the mid-Atlantic region of the U.S. through email addresses directly or listserv, e-newsletter, or mass email; Cross-sectional	i. Lack of bike lanes (Route-related; P) ii. Lack of walking/biking paths (Route-related; P) iii. Lack of sidewalks (Route-related; P) iv. Traffic volume (Safety; P) v. Crime level (Safety; P) vi. Difficult terrain (Safety; P) vii. Travel time to home (Destination-related; P)	a. Active commuting to work (Transport PA; R)	i-a. N ii-a. N iii-a. N iv-a. N v-a. N vi-a. N vii-a. N	Age, number of chronic diseases, perceived health status, self-efficacy, and perceived behavioral control
Bopp (2014) [39]	997 employed adults not working from home, USA	A convenience sample was recruited in medium-large cities in the mid-Atlantic region of the U.S. through email addresses directly or listserv, e-newsletter, or mass email; Cross-sectional	i. Lack of bike lanes (Route-related; P) ii. Lack of walking/biking paths (Route-related; P) iii. Lack of sidewalks (Route-related; P) iv. Traffic volume (Safety; P) v. Crime level (Safety; P) vi. Difficult terrain (Safety; P) vii. Travel time to home (Destination-related; P)	a. Active commuting to and from work (Transport PA; R)	Older adults i-a. N ii-a. N iii-a. N iv-a. N v-a. NR vi-a. N vii-a. – Younger adults i-a. N ii-a. N iii-a. N iv-a. N v-a. N vi-a. N vii-a. N	Sex, BMI, number of children, number of chronic diseases, number of cars in the household, self-efficacy, perceived behavioral control, perceived health status, behavioral beliefs, marital status, ethnicity, income, education, employment, social support, and residential environment
Buehler (2012) [30]	5091 employed adults, USA	A national survey to recruit a random sample of address list-based households; Cross-sectional	i. Distance to home (Destination-related; P)	a. Biking to work (Transport PA; R)	i-a. –	Sex, age, ethnicity, household income, access to cars/bikes, residential population density, residential area, bikeway supply, season, and workplace policies
Carlson (2018) [62]	1085 employed adults not working from home, USA	Employees were selected randomly from households systematically selected to vary in land use patterns and income; Cross-sectional	i. Land use mix (Destination-related; P) ii. Street connectivity (Route-related; P) iii. Walking/cycling facilities (Route-related; P) iv. Aesthetics; (Aesthetics; P) v. Traffic safety (Safety; P) vi. Pedestrian safety (Safety; P) vii. Crime safety (Safety; P)	a. Total active transport (Transport PA; R) b. Active transport around work (Transport PA; R) c. Active transport to/from work (Transport PA; R) d. Total MVPA (Total PA; O)	i-a. +; i-b. +; i-c. +; i-d. N ii-a. N; ii-b. +; ii-c. N; ii-d. N iii-a. N; iii-b. N; iii-c. +; iii-d. N iv-a. N; iv-b. N; iv-c. N; iv-d. N v-a. N; v-b. +; v-c. +; v-d. N vi-a. N; vi-b. +; vi-c. N; vi-d. + vii-a. –; vii-b. –; vii-c. N; vii-d. +	Sex, age, education, ethnicity, vehicles per adult, marital status, people per household, time at address, city, and clustering of participants within block groups
Christiansen (2017) [56]	4764 employed adults, Norway	A national survey was randomly sampled among residents in each county in Norway; Cross-sectional	i. Distance to home (Destination-related; P) ii. Limited parking availability (Destination-related; P) iii. Land use mix (Destination-related; O) iv. Distance to the city centre (Destination-related; O)	a. Trip from home to work by car (Transport SB; R)	i-a. + ii-a. – iii-a. – iv-a. +	Age, education, household income, and residential environment
Clark (2016) [51]	15,200 employed English adults, UK	A national survey of multistage sampling and the same individuals are re-interviewed in each wave; Longitudinal (follow-up: 1 year)	i. Distance to home (Destination-related; P) ii. Change in the distance to home between wave 1 and wave 2 (Destination-related; P)	a. Car commuting to work (Transport SB; R) b. Active commuting to work (Transport PA; R) c. Commute mode switch from car to non-car (Transport PA; R) d. Commute mode switch from non-car to car (Transport SB; R) e. Commute mode switch from active to non-active (Transport SB; R) f. Commute mode switch from non-active to active (Transport PA; R)	i-a. +; i-b. –; i-c. NR; i-d. NR; i-e. NR; i-f. NR ii-a. NR; ii-b. NR; ii-c. –; ii-d.+; ii-e. +; ii-f. –	Sex, age, education, employment type, household income, attitudes, household car ownership, current driving license, and residential environment (for a-b) Sex, age, education, employment type, household income, attitudes, household car ownership, current driving license, residential environment, and change in life events (for c-f)
Dalton (2013) [32]	1124 employed adults, UK	Recruited employees working in Cambridge through workplaces; Cross-sectional	i. Distance to the nearest bus stop (Destination-related; O) ii. Distance to the nearest railway station (Destination-related; O) iii. Number of bus stops (Destination-related; O) iv. Number of destinations in working area (Destination-related; O) v. Distance to home (Destination-related; O)	a. Public transport use to work (Transport PA; R) b. Biking to work (Transport PA; R) c. Walking to work (Transport PA; R)	i-a. N; i-b. N; i-c. N ii-a. N; ii-b. N; ii-c. N iii-a. N; iii-b. N; iii-c. N iv-a. +; iv-b. +; iv-c. N v-a. N; v-b. –; v-c. –	Sex, age, limiting illness, deprivation, education, children in household, car ownership, type of work, residential environment, and car parking availability
de Geus (2008) [19]	343 employed Flemish adults not working from home, Belgium	Recruited employees via newsletter distributed in Flanders and contacted local cycle communities for having enough cyclists; Cross-sectional	i. Traffic danger (Safety; P) ii. Bicycle lanes (Route-related; P) iii. Crime rate (Safety; P)	a. Cycling for transport (Transport PA; R)	i-a. N ii-a. N iii-a. N	Education
Forsyth (2014) [40]	446 employed adults not working from home, USA	A randomly selected sample of a residential area at first, and all households were invited in the second stage; Cross-sectional	i. Housing density (Destination-related; O) ii. Access points (Destination-related; O) iii. Percentage of commercial land use (Destination-related; O)	a. Travel PA (Transport PA; O) b. Leisure PA (Recreational PA; O) c. Total PA (Total PA; O)	i-a. +; i-b. N; i-c. + ii-a. N; ii-b. N; ii-c. N iii-a. +; iii-b. N; iii-c. N	Sex, age, ethnicity, education, marital status, housing tenure, household income, household size, PA at work, and neighbourhood clustering
Gehrke (2017) [57]	655 employed adults, USA	A national survey randomly sample household within Oregon and invite to participate through mail and telephone; Cross-sectional	i. Activity density (Destination-related; O) ii. Employment density (Destination-related; O) iii. Population density (Destination-related; O) iv. Retail density (Destination-related; O) v. Urban living infrastructure density (Destination-related; O) vi. Employment entropy (2 types of calculation) (Destination-related; O) vii. Employment-population balance (3 types of calculation) (Destination-related; O) viii. Block area (Destination-related; O) ix. Block density (2 types of calculation) (Destination-related; O) x. Connected node ratio (Route-related; O) xi. Connectivity index (4 types of calculation) (Route-related; O) xii. Cul-de-sac density (Route-related; O) xiii. Intersection density (Route-related; O) xiv. Intersection-Cul-de-sac ratio (Route-related; O) xv. The proportion of local roads (Route-related; O) xvi. The proportion of primary roads (Route-related; O) xvii. The proportion of secondary roads (Route-related; O) xviii. Street network density (Route-related; O)	a. Work-based walking (Transport PA; R)	i-a. N ii-a. N iii-a. + iv-a. N v-a. N vi-a. –, N vii-a. N, N, N viii-a. N ix-a. +, N x-a. N xi-a. N, N, N, N xii-a. N xiii-a. N xiv-a. N xv-a. N xvi-a. N xvii-a. N xviii-a. N	Sex, employment, and private vehicle ownership
Hamre (2014) [41]	4630 full-time employed adults, USA	A national survey to recruit a random sample of address list-based households; Cross-sectional	i. Distance to home (Destination-related; P)	a. Public transport use to work (Transport PA; R) b. Walking to work (Transport PA; R) c. Cycling to work (Transport PA; R)	i-a. N; i-b. –; i-c. –	Sex, age, ethnicity, household income, access to cars/bikes, residential population density, residential area, transit access, bikeway supply, season, and worksite policies
Handy (2011) [25]	420 employed adults not working from home, USA	A random sample of residents for each of the six communities in the U.S.; Cross-sectional	i. Distance to home (Destination-related; P) ii. Dangerous for bicycling (Safety; P)	a. Commuting to work by bicycle (Transport PA; R)	i-a. – ii-a. N	Sex, housing tenure, biking comfort, commuting beliefs and preference
Heinen (2013) [33]	4171 employed adults, Netherlands	Recruited employees of large organisations and residents of working age in Delft and Zwolle; Cross-sectional	i. Distance to home (Destination-related; O)	a. Cycling to work (Transport PA; R)	i-a. –	Sex, age, ethnicity, access to cars/scooters/bikes, purpose to use, sampling area, attitude, social support, facilities at work, and work policies
Karusisi (2014) [42]	4127 employed adults, France	Employees were recruited from a free medical check-up offered by National Health Insurance System; Cross-sectional	i. Destinations density around the workplace (Destination-related; O)	a. Walking for transport (Transport PA; R)	i-a. +	Sex, age, marital status, education, occupation, homeownership, perceived financial strain, household income, and the level of human development of the country of birth
Li (2018) [15]	2843 employed adults, Japan	A prospective cohort study of local government workers in a central part of Japan; Cross-sectional	i. Walkability (Composite index; O) ii. Number of parks/green spaces (Destination-related; O) iii. Number of sports facilities (Destination-related; O)	a. Habitual walking during leisure-time (Recreational PA; R) b. Habitual exercise during leisure-time (Recreational PA; R)	Men i-a. N; i-b. N ii-a. N; ii-b. N iii-a. N; iii-b. N Women i-a. N; i-b. N ii-a. N; ii-b. N iii-a. N; iii-b. N	Age, education, marital status, office worker, BMI, smoking status, alcohol consumption, sleeping hours, eating breakfast every day, depression, history of hypertension or diabetes, residential environment
Lucove (2007) [17]	987 employed adults, USA	A random sample selected from residential household phone numbers; Cross-sectional	i. A safe place to walk outside work (Safety; P)	a. Any leisure-time PA (Recreational PA; R) b. Work-break PA (Recreational PA; R) c. Overall PA (Total PA; R)	i-a. N; i-b. N; i-c. N	Sex, age, ethnicity, education, physical disability, and general health
Macdonald (2019) [64]	513 employed adults, UK	A random sample selected from the electoral roll within local authority; Cross-sectional	i. Access to public PA facilities (Destination-related; O) ii. Access to private PA facilities (Destination-related; O)	a. PA (Total PA; R)	i-a. N ii-a. N	Sex, age, and income deprivation
Mackenbach (2016) [52]	482 employed adults, New Zealand	A national survey of multistage stratified sampling; Cross-sectional	i. Population density (Destination-related; O) ii. Housing density (Destination-related; O) iii. Apartment density (Destination-related; O) iv. Job accessibility (Destination-related; O) v. Land use mix (Destination-related; O) vi. Number of bus stops (Destination-related; O) vii. Number of rail stations (Destination-related; O)	a. Active commuting to work (Transport PA; R)	i-a. N ii-a. – iii-a. N iv-a. + v-a. + vi-a. N vii-a. +	Sex, age, income, household type, season, day of the week, and trip distance
Marquet (2018) [63]	147 full-time employed women not working from home, USA	A convenience sample of women living in the U.S; Cross-sectional	i. Walkability (Composite index; O) ii. Walk Score (Composite index; O) iii. Vegetation index (Aesthetics; O)	a. Total MVPA while at work (Occupational PA; O) b. Total MVPA around the workplace (Occupational PA; O)	i-a. +; i-b. + ii-a. N; ii-b. N iii-a. –; iii-b. –	Age, having children, income, work–home distance, amount of non-work PA
Marquet (2019) [14]	119 employed adults, USA	A multistage sampling frame was used to select adults form list-assisted telephone random-digit-dialing methods randomly; Cross-sectional	i. Perceived walkability (Composite index; P) ii. Walkability (Composite index; O) iii. Walk Score (Composite index; O) iv. Vegetation index (Aesthetics; O)	a. Active minutes at work (Occupational PA; O)	i-a. + ii-a. N iii-a. + iv-a. N	Sex, BMI, income, work type, residential walkability, outside work PA
Merom (2008) [20]	794 employed adults not working from home, Australia	A random sample selected from electric telephone white pages; Longitudinal (follow-up: 1 month)	i. Distance to home (Destination-related; P)	a. Single-day active commuting to work (Transport PA; R) b. Usual active commuting to work (Transport PA; R)	i-a. –; i-b. N	Age, education, marital status, BMI, self-efficacy, active commuting beliefs, and total PA
Panter (2011) [26]	1164 employed adults, UK	Recruited employees working in Cambridge through workplaces; Cross-sectional	i. Public transport (Destination-related; P) ii. Little traffic (Safety; P) iii. Routes for walking (Route-related; P) iv. Safe to cross the road (Safety; P) v. Dangerous for cyclists (Safety; P) vi. Routes for cycling (Route-related; P) vii. Distance to home (Destination-related; P)	a. Walking to work (Transport PA; R) b. Cycling to work (Transport PA; R)	With car availability in household i-a. +; i-b. NR ii-a. –; ii-b. N iii-a. N; iii-b. NR iv-a. N; iv-b. N v-a. NR; v-b. N vi-a. NR; vi-b. + vii-a. –; vii-b. NR Without car availability in household i-a. N; i-b. NR ii-a. N; ii-b. N iii-a. N; iii-b. NR iv-a. N; iv-b. N v-a. NR; v-b. N vi-a. NR; vi-b. N vii-a. –; vii-b. NR	Sex, current driving licence, and attitude of car use (for a) Sex, education, weight status, limiting illness, number of children, car ownership, and attitude of car use (for b)
Panter (2011) [27]	1279 employed older adults, UK	A prospective cohort of adults who registered at 121 General Practices within Norwich and surrounding towns; Cross-sectional	i. Route-length ratio (Route-related; O) ii. Main or secondary road on the route (Route-related; O) iii. Land use mix (Destination-related; O) iv. Density of road traffic accidents (Safety; O) v. Distance to home (Destination-related; O)	a. Active commuting to work (Transport PA; R)	Men i-a. N ii-a. NR iii-a. NR iv-a. N v-a. – Women i-a. NR ii-a. – iii-a. NR iv-a.NR v-a. –	Age, social class, BMI, habit for walking or cycling for transport, and residential urban-rural status, and residential road density
Panter (2013) [34]	419 employed car commuters to work, UK	Recruited employees working in Cambridge through workplaces; Cross-sectional	i. Distance to home (Destination-related; P) ii. Supportive environment (Composite index; P)	a. Incorporating walking or cycling into car journeys to work (Transport PA; R)	i-a. N ii-a. +	BMI, work type, deprivation, workplace car parking, attitude towards car, social norm, and habit strength for car use
Panter (2013) [35]	655 employed adults, UK	Recruited employees working in Cambridge through workplaces; Longitudinal (follow-up: 1 year)	i. Distance to home (Destination-related; P) ii. Destinations within walking distance (Destination-related; O) iii. Public transport (Destination-related; P) iv. Little traffic (Safety; P) v. Walking routes (Route-related; P) vi. Safe to cross the road (Safety; P) vii. Dangerous for cyclists (Safety; P) viii. Cycling routes (Route-related; P)	a. Uptake of walking (Transport PA; R) b. Uptake of cycling (Transport PA; R) c. Uptake of alternatives to the car (Transport PA; R) d. Maintenance of walking (Transport PA; R) e. Maintenance of cycling (Transport PA; R) f. Maintenance of alternatives to the car (Transport PA; R)	i-a. N; i-b. N; i-c. N; i-d. N; i-e. N; i-f. N ii-a. N; ii-b. N; ii-c. N; ii-d. N; ii-e. N; ii-f. N iii-a. +; iii-b. NR; iii-c. N; iii-d. N; iii-e. NR; iii-f. N iv-a. N; iv-b. N; iv-c. N; iv-d. N; iv-e. N; iv-f. N v-a. N; v-b. NR; v-c. N; v-d. N; v-e. NR; v-f. N vi-a. N; vi-b. N; vi-c. N; vi-d. N; vi-e. N; vi-f. N vii-a. NR; vii-b. N; vii-c. N; vii-d. NR; vii-e. N; vii-f. N viii-a. NR; viii-b. +; viii-c. +; viii-d. NR; viii-e. N; viii-f. N	Sex, age, weight status, education, number of children, housing tenure, home location, area-level deprivation, residential environment, attitude to use car, perceived behaviour control, social norm, habit strength, and workplace car parking
Panter (2014) [43]	655 employed adults, UK	Recruited employees working in Cambridge through workplaces; Longitudinal (follow-up: 1 year)	i. Public transport (Destination-related; P) ii. Little traffic (Safety; P) iii. Walking routes (Route-related; P) iv. Safe to cross the road (Safety; P) v. Dangerous for cyclists (Safety; P) vi. Cycling routes (Route-related; P)	a. Change in time spent walking on the commute (Transport PA; R) b. Change in time spent cycling on the commute (Transport PA; R) c. Change in percentage of car-only trips on the commute (Transport SB; R) d. Uptake of walking on the commute (Transport PA; R) e. Uptake of cycling on the commute (Transport PA; R) f. Uptake of an alternative to the car on the commute (Transport PA; R)	i-a. N; i-b. NR; i-c. N; i-d. N; i-e. NR; i-f. + ii-a. N; ii-b. N; ii-c. N; ii-d. N; ii-e. N; ii-f. N iii-a. N; iii-b. NR; iii-c. N; iii-d. N; iii-e. NR; iii-f. N iv-a. N; iv-b. N; iv-c. –; iv-d. N; iv-e. N; iv-f. N v-a. NR; v-b. N; v-c. +; v-d. NR; v-e. N; v-f. – vi-a. NR; vi-b. N; vi-c. N; vi-d. NR; vi-e. N; vi-f. N	Sex, age, education, season, housing tenure, household composition, access to cars/bikes, current driving licence, and limiting illness
Paul (2019) [65]	23,231 full-time employed adults working in the U.S. Department of the Interior, USA	Employees working in the U.S. Department of the Interior were emailed an invitation to participate and hyperlink to the survey; Cross-sectional	i. Distance to home (Destination-related; O) ii. Workplace located in a non-metro area (Destination-related; O)	a. Commuting to work by walking (Transport PA; R) b. Commuting to work by cycling (Transport PA; R) c. Commuting to work by non-active mode incorporating walking/cycling (Transport PA; R)	i-a. –; i-b. –; i-c. N ii-a. +; ii-b. –; ii-c. –	Sex, age, and residential environment
Piatkowski (2015) [46]	2030 employed bicycling commuters, USA	Participants were drawn from individuals that sign-up to receive more information about “Bike to Work Day” (BTWD) online and solicited via email to participate; Cross-sectional	i. Distance to home (Destination-related; P) ii. Street link-to-node ratio (Route-related; O) iii. Intersection density (Route-related; O) iv. Safety and infrastructure (Composite index; P) v. Relative convenience (Composite index; P)	a. Biking to work on BTWD (Transport PA; R) b. Occasional commuter (Transport PA; R)	i-a. –; i-b. –; ii-a. N; ii-b. N iii-a. N; iii-b. N iv-a. N; iv-b. N v-a. –; v-b. –	Sex, age, ethnicity, household size, education, household income, car availability, attitude and perception factors, and residential environment
Pritchard (2019) [66]	195 employed adults, Norway	A fixed sample which the same group of participants working in intra-city workplaces responded to both surveys; Cross-sectional	i. Distance to home (Destination-related; O)	a. Commuting to work by public transport (Transport SB; R) b. Commuting to work by car/motorcycle (Transport SB; R)	i-a. +; i-b. +	Access to car/bicycle, having children, and paid parking around workplace
Prodaniuk (2004) [16]	897 employed adults, Canada	Employees working in three large organisations located in Western Canada were sent a research invitation within the internal mail system of the workplace; Cross-sectional	i. Perceived Workplace Environment Scale (Composite index; P)	a. Workplace PA (Occupational PA; R) b. Leisure-time PA (Recreational PA; R)	i-a. +; i-b. +	Workplace
Quinn (2017) [58]	111,808 employed adults, USA	A national survey of random sampling using a telephone survey of landline numbers; Cross-sectional	i. Distance to home (Destination-related; P) ii. Travel time to home (Destination-related; P)	a. Commuting to work by walking (Transport PA; R) b. Commuting to work by cycling (Transport PA; R)	i-a. –; i-b. – ii-a. –; ii-b. –	Sex, age, ethnicity, education, household income, and geographic location
Rafferty (2016) [53]	26 full-time office workers, UK	A convenience sample of employees at Glasgow Caledonian University recruited by email; Cross-sectional	i. Distance to home (Destination-related; O)	a. Number of steps (Total PA; O) b. Total time spent in MVPA (Total PA; O) c. Steps were taken during the commute (Transport PA; O) d. Time spent in MVPA during the commute (Transport PA; O)	i-a. N; i-b. N; i-c. N; i-d. N	NA
Schoner (2015) [47]	614 employed adults, USA	Sample of residents from five corridors in the U.S.; Cross-sectional	i. Distance to home (Destination-related; O)	a. Participation in bicycle commuting to work (Transport PA; R)	i-a. –	Age, employment, residential preference, travel attitudes, and residential environment
Schwartz (2009) [21]	117 employed adults, USA	Convenience sample selected from 1 zone in Maryland; Cross-sectional	i. Without cul-de-sacs (Route-related; P) ii. Four-way intersections (Route-related; P) iii. Sidewalks (Route-related; P) iv. Bicycle or pedestrian trails (Route-related; P) v. Trees along the streets (Aesthetics; P) vi. Free from litter (Aesthetics; P) vii. Traffic danger (Safety; P) viii. Crosswalks and pedestrian signals (Safety; P)	a. Total number of walking trips taken from the workplace (Transport PA; O) b. Steps were taken at or near work (Occupational PA; O) c. Average weekday steps (Total PA; O)	i-a. +; i-b. N; i-c. N ii-a. N; ii-b. N; ii-c. N iii-a. +; iii-b. N; iii-c. N iv-a. N; iv-b. N; iv-c. N v-a. N; v-b. N; v-c. N vi-a. +; vi-b. N; vi-c. N vii-a. N; vii-b. N; vii-c. N viii-a. +; viii-b. N; viii-c. N	NA
Troped (2010) [23]	87 employed adults, USA	A fixed follow-up sample limited to trail users; Cross-sectional	i. Intersection density (Route-related; O) ii. Land use mix (Destination-related; O) iii. Residential population density (Destination-related; O) iv. Housing unit density (Destination-related; O) v. Vegetation index (Aesthetics; O)	a. MVPA within 1 km of the workplace (Occupational PA; O)	i-a. N ii-a. N iii-a. + iv-a. + v-a. N	Sex, age, ethnicity, and education
Umstattd (2011) [28]	173 university employees, USA	A convenience sample of university employees; Cross-sectional	i. Worksite Supportive Environments for Active Living Surveys (Composite index; P)	a. MVPA (Total PA; R)	i-a. N	Sex, age, ethnicity, health status, position type, self-regulation, self-efficacy, and social support
Watts (2013) [36]	48,916 employed Canadian adults not working from home, Canada	A multistage sampling frame was used to select households across Canada randomly; Cross-sectional	i. Access to PA amenities (Composite index; P)	a. Leisure-time PA (Recreational PA; R)	i-a. +	Sex, age, income, and education
Watts (2016) [54]	1538 employed young adults, USA	Employees were recruited from the third wave of a 10-year longitudinal study in young people who progressed from adolescence to young adulthood; Cross-sectional	i. Distance to fitness facilities (Destination-related; P) ii. Distance to home (Destination-related; P)	a. MVPA (Total PA; R) b. Time spent in walking or biking to get places (Transport PA; R)	i-a. N; i-b. NR ii-a. N; ii-b. –	Age, ethnicity, and socio-economic status
Wen (2010) [24]	888 employed parents not working from home, Australia	Employed parents of students studying in public primary schools located in the inner west of Sydney were recruited; Cross-sectional	i. Public transport (Destination-related; P) ii. Car parking (Destination-related; P) iii. Reputation for a safe place (Safety; P) iv. Distance to home (Destination-related; P)	a. Travel to work by car (Transport SB; R)	i-a. – ii-a. + iii-a. N iv-a. N	Clustering by the school and the within-school intraclass correlation for travel to work by car
Yang (2015) [48]	1332 employed adults not working from home, USA	Multistage stratified sampling using list-assisted telephone random-digit-dialling; Cross-sectional	i. Healthy restaurants (Destination-related; P) ii. Transit stop (Destination-related; P) iii. Sidewalks (Route-related; P) iv. Shops, stores, or markets (Destination-related; P) v. Facilities to bicycle (Route-related; P) vi. Recreation facilities (Destination-related; P) vii. Crime rate (Safety; P) viii. Dangerous traffic for pedestrian (Safety; P) ix. Distance to home (Destination-related; O)	a. Public transport use (Transport PA; R) b. Active commuting (Transport PA; R)	i-a. N; i-b. N ii-a. N; ii-b. N iii-a. N; iii-b. N iv-a. N; iv-b. N v-a. N; v-b. N vi-a. N; vi-b. + vii-a. N; vii-b. N viii-a. N; viii-b. N ix-a. N; ix-b. –	Sex, age, BMI, household car ownership, and education (for a) Sex, age, BMI, and household car ownership (for b)
Yang (2017) [59]	2757 employed adults, UK	Recruited employees registered at 121 General Practices within Norwich and surrounding towns; Longitudinal (follow-up: 7 years)	i. Distance to home (Destination-related; O) ii. Route length ratio (Route-related; O) iii. Main road on the route (Route-related; O) iv. Secondary road on route (Route-related; O) v. Main or secondary road along route (Route-related; O) vi. Number of streetlights along route (Safety; O) vii. Land use mix (Destination-related; O) viii. Density of road traffic accidents (Safety; O) ix. Density of fatal and serious road traffic accidents (Safety; O)	a. Uptake of active commuting (Transport PA; R) b. Maintenance of active commuting (Transport PA; R)	i-a. –; i-b. – ii-a. N; ii-b. N iii-a. N; iii-b. N iv-a. N; iv-b. N v-a. –; v-b. – vi-a. +; vi-b. NR vii-a. N; vii-b. N viii-a. N; viii-b. N ix-a. N; ix-b. N	Sex, age, BMI, and residential environment
Zhang (2019) [67]	98 employed Chinese adults, China	A convenience sample recruited from two-night schools offered by two universities in Beijing and Shanghai, China; Longitudinal (follow-up: 1 month)	i. Residential density (Destination-related; P) ii. Land-use diversity (Destination-related; P) iii. Land-use accessibility (Destination-related; P) iv. Street connectivity (Route-related; P) v. Aesthetics (Aesthetics; P) vi. Walking infrastructure (Route-related; P) vii. Traffic safety (Safety; P) viii. Crime safety (Safety; P)	a. Transport-related cycling at time 1 (Transport PA; R) b. Transport-related cycling at time 2 (Transport PA; R)	i-a. N; i-b. N ii-a. N; ii-b. N iii-a. N; iii-b. N iv-a. N; iv-b. N v-a. N; v-b. N vi-a. N; vi-b. N vii-a. N; vii-b. N viii-a. N; viii-b. N	NA
Zhang (2019) [68]	157 employed Chinese adults, China	A convenience sample recruited from two-night schools offered by two universities in Beijing and Shanghai, China; Longitudinal (follow-up: 1 month)	i. Walkability (Composite index; P)	a. Transport-related walking at time 1 (Transport PA; R) b. Transport-related walking at time 2 (Transport PA; R)	i-a. N; i-b. N	Sex, age, marital status, education, number of children, BMI, income level, and general health

Note: *PA* Physical activity, *SB* Sedentary behaviour, *O* Objectively-measured, *P* Perceived, *R* Reported, + Positive association, *N* Non-significant association, − Negative association, *NR* Not reported, *NA* Not applicable, *BMI* Body mass index, *MVPA* Moderate-to-vigorous physical activity

All of the studies reviewed sampled working adults while some studies examined the associations in question for particular population subgroups such as women [38, 63], parents [24, 50], specific nationalities [19, 36, 51, 67, 68], commuters [34, 46], and workers working in an university [28] and local governments [65]. Sample sizes ranged from 26 to 111,808, of which three had a sample size lower than 100 [23, 53, 67] and five with a sample size larger than 10,000 [36, 51, 58, 61, 65].

Among the 55 studies reviewed, most investigated physical activity (*n* = 52) rather than sedentary behaviour (*n* = 7); four investigated both physical activity and sedentary behaviour. The transport domain was most commonly examined in relation to both physical activity (40 out of 52 studies) and sedentary behaviour (7 out of 7 studies). Nearly half of the studies used validated outcome measures, including accelerometers [14, 21, 23, 40, 53, 60, 62, 63] and questionnaires with acceptable reliability and validity [16, 17, 21, 26–28, 31, 32, 34, 36, 37, 44, 45, 49, 50, 54, 61, 64, 67, 68]. Notably, 28 studies assessed self-reported physically-active and sedentary behaviours without reporting the reliability or validity of the questions or questionnaires used.

More than half of the studies measured perceived built environments (*n* = 29, 52.7%), 34.5% of them measured environments objectively, and 12.7% included both types of measure in their studies. A vague boundary (e.g., near or surrounding the workplace) was the most commonly used as a perceived neighbourhood definition. When buffers were applied to define workplace neighbourhoods, a 400- [40, 57, 63] or 800-m radius [14, 40, 45, 64] and the network buffer [18, 23, 40, 46, 64] were the most frequently used buffer size and type, respectively.

### Built environment correlates of physical activity and sedentary behaviour

Detailed syntheses of the findings are shown in Table 2. We reported the findings according to “instances” rather than “studies” as most of the studies reported associations of different built environment attributes with multiple domains of physical activity and sedentary behaviours. Overall, there were 455 instances in our analysis, nearly half of instances involved destination-related attributes (193 out of 455), followed by safety (111 out of 455) and route-related attributes (105 out of 455). Additionally, most of them examined physical activity (431 out of 455), particularly within the transport domain (325 out of 431). Only 24 out of 455 examined sedentary behaviour, and all of them focused on transport settings.

**Table 2:** Workplace neighbourhood built environment attributes and workers’ active and sedentary behaviours: summary of instances

Workplace neighbourhood built environment attributes	Physical activity and sedentary behavior	Good			Fair			Poor			Total			
		+	N	–	+	N	–	+	N	–	+	N	–	Sum
Composite environmental indices	Physical activity													
	Total	0	1	0	0	1	0	0	0	0	0	2	0	2
	Occupational	2	2	0	3	1	0	0	0	0	5	3	0	8
	Transport	0	4	2	2	0	0	0	0	0	2	4	2	8
	Recreational	5	1	0	1	4	0	0	0	0	6	5	0	11
	(sub-total)	7	8	2	6	6	0	0	0	0	13	14	2	29
	Sedentary behaviour													
	Total	0	0	0	0	0	0	0	0	0	0	0	0	0
	Occupational	0	0	0	0	0	0	0	0	0	0	0	0	0
	Transport	0	0	0	0	0	0	0	0	0	0	0	0	0
	Recreational	0	0	0	0	0	0	0	0	0	0	0	0	0
	(sub-total)	0	0	0	0	0	0	0	0	0	0	0	0	0
Route-related attributes	Physical activity													
	Total	0	7	0	2	0	0	0	0	0	2	7	0	9
	Occupational	0	5	0	1	1	0	0	0	0	1	6	0	7
	Transport	10	51	2	2	17	1	0	0	0	12	68	3	83
	Recreational	0	1	0	1	1	0	0	0	0	1	2	0	3
	(sub-total)	10	64	2	6	19	1	0	0	0	16	83	3	102
	Sedentary behaviour													
	Total	0	0	0	0	0	0	0	0	0	0	0	0	0
	Occupational	0	0	0	0	0	0	0	0	0	0	0	0	0
	Transport	0	2	0	0	1	0	0	0	0	0	3	0	3
	Recreational	0	0	0	0	0	0	0	0	0	0	0	0	0
	(sub-total)	0	2	0	0	1	0	0	0	0	0	3	0	3
Destination-related attributes	Physical activity													
	Total	2	9	0	1	3	0	0	2	0	3	14	0	17
	Occupational	2	1	0	0	4	0	0	0	0	2	5	0	7
	Transport	37	55	2	22	17	2	0	2	0	59	74	4	137
	Recreational	0	5	0	2	10	0	0	0	0	2	15	0	17
	(sub-total)	41	70	2	25	34	2	0	4	0	66	108	4	178
	Sedentary behaviour													
	Total	0	0	0	0	0	0	0	0	0	0	0	0	0
	Occupational	0	0	0	0	0	0	0	0	0	0	0	0	0
	Transport	0	1	4	2	1	7	0	0	0	2	2	11	15
	Recreational	0	0	0	0	0	0	0	0	0	0	0	0	0
	(sub-total)	0	1	4	2	1	7	0	0	0	2	2	11	15
Safety	Physical activity													
	Total	2	4	0	0	2	0	0	0	0	2	6	0	8
	Occupational	0	2	0	1	1	0	0	0	0	1	3	0	4
	Transport	7	70	4	0	8	0	0	0	0	7	78	4	89
	Recreational	0	2	0	0	2	0	0	0	0	0	4	0	4
	(sub-total)	9	78	4	1	13	0	0	0	0	10	91	4	105
	Sedentary behaviour													
	Total	0	0	0	0	0	0	0	0	0	0	0	0	0
	Occupational	0	0	0	0	0	0	0	0	0	0	0	0	0
	Transport	0	2	2	0	2	0	0	0	0	0	4	2	6
	Recreational	0	0	0	0	0	0	0	0	0	0	0	0	0
	(sub-total)	0	2	2	0	2	0	0	0	0	0	4	2	6
Aesthetics	Physical activity													
	Total	0	3	0	0	0	0	0	0	0	0	3	0	3
	Occupational	0	3	2	0	1	0	0	0	0	0	4	2	6
	Transport	1	7	0	0	0	0	0	0	0	1	7	0	8
	Recreational	0	0	0	0	0	0	0	0	0	0	0	0	0
	(sub-total)	1	13	2	0	1	0	0	0	0	1	14	2	17
	Sedentary behaviour													
	Total	0	0	0	0	0	0	0	0	0	0	0	0	0
	Occupational	0	0	0	0	0	0	0	0	0	0	0	0	0
	Transport	0	0	0	0	0	0	0	0	0	0	0	0	0
	Recreational	0	0	0	0	0	0	0	0	0	0	0	0	0
	(sub-total)	0	0	0	0	0	0	0	0	0	0	0	0	0

Note: + Positive association, *N* Non-significant association, − Negative association

#### Composite environmental indices

There were 14 instances where an association was estimated between a workplace neighbourhood composite index and a physical activity outcome. All measured walkability (primarily based on density, land use mix, and street connectivity), with the only exception measuring the presence of facilities and routes for walking through an audit tool [37]. Most of the instances regarding walkability were measured objectively by geographic information systems based on established indices (*n* = 7) and Walk Score® (*n* = 3) while the remainder measured perceived walkability (n = 3). Of 14 instances where composite indexes were examined, only four showed positive associations with physical activity [14, 63]; all of which were in relation to the occupational domain. More than 70% of the instances reported were non-significant.

Fifteen instances used composite indices mixing other attributes (e.g., organised sports teams and classes within the workplace) in addition to workplace neighbourhood built environment attributes. The majority of the instances (*n* = 9) found positive associations with physical activity, mostly about the recreational domain. The remainder of instances found either non-significant [28, 46, 61] or negative associations [46] in relation to physical activity.

There were no instances of associations between composite indices and sedentary behaviour.

#### Route-related attributes

Most of the instances examined routes for pedestrians or cyclists and street connectivity, as route-related attributes to investigate their associations with physical activity, especially in the transport settings, which accounted for 81% of the instances. Around 16% of the 102 instances reported positive associations with physical activity [18, 21, 26, 35, 44, 49, 62]. The majority of instances (*n* = 83) were non-significant. However, three instances were negatively associated with physical activity; all of which were in relation to the transport domain [27, 59].

All three instances of examining an association between route-related attributes and sedentary behaviour found that routes for pedestrians or cyclists were not associated with sedentary transport behaviour [29, 43].

#### Destination-related attributes

The majority of the instances used the presence, density, and diversity of destinations as destination-related attributes in the workplace neighbourhood to examine their associations with physical activity, especially during transport-related contexts, which accounted for 77% of the instances. Almost 40% of the 178 instances which examined destination-related attributes were found to be positively associated with physical activity, mostly in relation to the transport domain. Over 60% of the instances were non-significant. Additionally, four instances were found to be negatively associated with transport physical activity [22, 52, 57, 65]; of which one negative association examined car parking with transport physical activity [22]. The features of these destination-related attributes identified in the positive and negative associations were different. The presence or density of shops, transport stops, and recreational facilities were more identified in the positive associations; by contrast, all of the negative associations identified that longer distance between workplace and home and car parking around workplace were associated with lower levels of transport physical activity.

The majority of instances (11 out of 15) reported negative associations between destinations-related attributes and sedentary behaviour; all of which were regarding the transport domain [24, 29, 50, 51, 56, 66]. Most of these destination-related attributes examined were the distance between workplace and home. Furthermore, the only two instances examining car parking showed positive associations with sedentary transport behaviour [24, 56]. The remainder of instances showed non-significant associations [24, 43]. The diversity of destination-related attributes examined in sedentary behaviour was less than in physical activity; a higher proportion of instances examined the distance between workplace and home with sedentary behaviour.

#### Safety

The instances in relation to workplace neighbourhood safety mainly measured the traffic (e.g., low volume of traffic for pedestrians and bicyclists) and crime safety (e.g., low crime rates). Less than 10% of the 105 instances of estimates between safety and physical activity showed positive associations [21, 44, 50, 59, 62]. Over 85% of the instances were not significant. Also, there were four instances where safety was negatively associated with transport-related physical activity [26, 43, 62].

Most of the instances (4 out of 6) regarding associations of safety with sedentary behaviour showed non-significant associations. The remainder of the instances found that perceiving it to be safer to cross the road and cycle was associated with a lower likelihood of car-only trips [43]. All of the instances were examined in the transport settings.

#### Aesthetics

All but 3 of the 17 instances examining associations between workplace neighbourhood aesthetics and physical activity were not significant. There was one instance of a positive association between perceptions that streets were free from litter and transport physical activity [21] and two instances of negative associations between objectively measured greenness and occupational physical activity [63].

None of the instances reported indicators of aesthetics with any domains of sedentary behaviour.

## Discussion

This review examined studies of the associations of workplace neighbourhood built environment attributes with active and sedentary behaviours among adults working in occupations involving prolonged indoor sitting. We found most of the instances examining associations between the workplace neighbourhood built environment and physical activity to be non-significant, irrespective of the study quality. Our findings are consistent with a previous review on workplace neighbourhood built environments [10]. This previous review also showed mostly non-significant associations of workplace neighbourhood built environment attributes with physical activity [10]. However, another systematic review on the residential neighbourhood reported consistently positive associations between accessibility to destinations and transport-related walking [12]. These findings suggest that compared with the residential environment, the workplace environment seems to be a less important context for supporting physically-active behaviours. A potential explanation for the weaker associations of workplace neighbourhood built environment attributes with physical activity in comparison with residential neighbourhoods may be due to workers having less autonomy over behavioural decisions during working hours, especially for those who perceive their managers disapprove of absenteeism from desks for walking [71]. Additionally, a lack of time for exercising during typical working hours may be another explanation for the weaker associations [15].

We found that destination-related attributes, notably longer distances between workplace and home, as well as better access to car parking around the workplace, were positively associated with transport-related sedentary behaviour; the sedentary transport behaviour in all the reviewed studies was travelling by car. A previous review on residential neighbourhood built environments found that better access to recreational facilities and public open spaces were negatively associated with transport-related sedentary behaviour [11]. Although varied destination-related attributes were measured in these two reviews, these findings suggest that better access to destinations, except for car parking, may be disincentives for transport-related sedentary behaviour, in both workplace and residential neighbourhoods. A short travelling distance to the workplace may make it more likely that workers undertake active commuting [72] which can replace time spent in car travel. For this reason, it may not matter whether there are well-maintained pathways or safe routes in or around the workplace neighbourhood if workers live too far from their workplaces to commute actively. Urban design policies on co-locating residential and workplaces together and decreasing car parking spaces around the workplace or moving car parking further away from workplaces may be effective strategies to reduce workers’ time spent in car travel.

The difference in the number of included studies between the previous review [10] and our review was mainly explained by the novel studies published after 2018 and additional studies examining specific neighbourhood built environment attributes. Our findings may suggest an authentic lack of an association between workplace built environments and physical activity, or the null findings may be attributable to the misperceptions of neighbourhood environments. A previous study reported that those who were less physically active for transport purposes perceived their high walkable residential neighbourhood as low walkable [73]. Such a misperception of neighbourhood environments may attenuate the associations of perceived workplace neighbourhood built environment attributes with active behaviours because most of the reviewed studies measured perceived environmental attributes.

Based on the review findings, there are some research priorities suggested for improving the quality of future relevant studies, as follows.

### Conducting research on workplace neighbourhood built environment and sedentary behaviour

Most of the previous studies examined the relationships of the workplace neighbourhood built environment with workers’ physical activity, rather than their sedentary behaviour. Considering the increased proportion of workers in desk-based occupations [74] and the high proportion of sitting time occurring in the workplace [1, 2], more attention is needed in investigating sedentary behaviour and workplace neighbourhood built environment correlates. A recent review showed that previous studies on workplace environments and sedentary behaviour mainly focused on the workplace interior environment, such as workstations [10]; however, our findings suggest that some destination-related attributes surrounding workplaces were associated with sitting time among workers. For developing effective approaches to improving workers’ health through reducing sitting time, studies on workplace neighbourhood built environments and sedentary behaviour, particularly the domains most likely to be affected (e.g., occupational and transport-related sedentary behaviour), are needed.

### Improving measurement and diversity of workplace neighbourhood built environment

Most previous studies assessed the workplace neighbourhood built environment using perceived measures; however, perceptions of the attributes of neighbourhoods could vary markedly between individuals, regardless of the objective environmental attributes that exist in the workplace neighbourhood. Furthermore, all the reviewed studies identified the shortest commuting route between workplace and home [27, 32, 33] irrespective of workers’ transport modes. The shortest commuting route may not represent the routes taken by the individuals. Future research included both perceived, and objective measures of the workplace neighbourhood built environment and monitoring the actual commuting routes (e.g., by global positioning system) for workers is encouraged to clarify their associations with active and sedentary behaviours. Additionally, some of the reviewed studies measured the variety of public open space in the workplace neighbourhood without considering the quality of such attributes. Previous research has shown that the quality of built environment attributes, including destinations such as parks, may be an essential factor to influence individuals’ active behaviours [75, 76] and thus should be considered. Future research on diverse varieties of public open space around the workplace and multiple measures for accessibility (e.g., the number of public transport stops as well as its frequency) may provide insights that will be relevant to developing effective strategies to promoting workers’ physical activity and reducing sedentary behaviour.

### Developing a framework for defining the influential buffers of workplace neighbourhood built environments for active and sedentary behaviours

Many of the reviewed studies did not clearly define the locations or areas of environmental attributes. Some studies used ambiguous terms (e.g., at or around the workplace) without specifying the neighbourhood’s boundary [22, 36, 77]. The core rationale for distinguishing interior and neighbourhood contexts of workplaces is its implications for where responsibility for improvements lies – i.e., land/property owners or governments. Furthermore, re-examining the influential buffers of the workplace is essential as it may be smaller than the frequently used buffers (e.g., 400- and 800-m) in research around residences [69] due to limited free time [15] and less autonomy over their behavioural decisions [71] for workers during working hours. Some other studies combined interior facilities and workplace policies [36, 37, 46] while assessing environmental attributes. These additional variables may contribute to stronger associations. Future studies are recommended to develop a framework in specifying the size of the workplace neighbourhood with clear definitions when examining their associations with physical activity or sedentary behaviours.

### Enhancing the correspondence between where built environments and behaviours are assessed

Most of the previous studies did not precisely designate ‘where’ the active or sedentary behaviours occurred, whereas they assessed built environment attributes surrounding the workplace only. The disparity between these variables may lead to a misinterpretation of workplace neighbourhood built environments due to the contribution of active or sedentary behaviours in non-work contexts to total physical activity. For example, workers who engaged in more light-intensity physical activity during working hours do compensate by doing less active during non-working hours [78]. Therefore, distinguishing the venue and different intensities for these behaviours in different contexts could inform detailed information to examine whether the compensation of physical activity or sedentary behaviour occurred. For ascertaining the associations of the workplace neighbourhood on physical activity or sedentary behaviour that occurs during work hours, or during commuting, studies which could identify specific venues (e.g., global positioning system) and timing-specific behaviours (e.g., accelerometer) are suggested.

### Considering potential confounding factors

When considering the workplace environmental correlates of physical activity or sedentary behaviour, some potential covariates should be examined in future research. For example, some geographic attributes attached to locations play an important role when individuals choose where to reside and work [79]. The self-selection of the residence and workplace may moderate the association of environmental attributes with active and sedentary behaviours. Referring to the ecological model, there may be an accumulative effect across different levels of factors. Individual motivations and attitudes, lifestyle preferences, social supports, interior workplace facilities, and workplace health promotion programs, may all contribute to the associations of workplace built environment attributes with workers’ physical activity and sedentary behaviour to some extent [45]. However, few of the reviewed studies accounted for factors such as the preference of the workplace while examining the associations in question. Studies considering these additional factors will provide additional evidence for the independent associations of environmental correlates with active or sedentary behaviours.

### Implementing research in diverse settings with prospective designs

Previous studies on workplace environmental correlates of active or sedentary behaviours have been mainly conducted in Western countries such as the USA and the UK. More relevant studies from non-Western countries should be encouraged because different countries or areas have varied behaviour patterns and neighbourhood built environments. For example, there are marked differences in the prevalence of active commuting across regions [80]. The low prevalence of physically-active commuting in Western countries leads to most of these studies investigating how to promote active commuting to or from work (i.e., transport physical activity) but less into other domains. However, studies giving weight to other domains of physical activity and sedentary behaviour may have more contributions to increase the total amount of physical activity and reduce the total time of sedentary behaviour, especially in countries or areas with a relatively high prevalence of active commuting to work. Additionally, future research should use prospective or experimental designs to evaluate whether changes in workplace neighbourhood built environments affect active and sedentary behaviours, rather than cross-sectional designs, which form the majority of the existing evidence base.

## Conclusions

Desk-based workers can spend around 80% of their working hours sedentary and can have limited opportunities for physical activity in and around the workplace. Synthesizing the current research evidence, we found that workers who lived further from their work and who could easily access car parking surrounding the workplace had a higher likelihood of transport-related sedentary behaviour. However, we found that workplace neighbourhood built environments such as route-related attributes, safety, and aesthetics did not appear to be influential for workers’ physically-active and sedentary behaviours. Designing mixed-use neighbourhoods where there are opportunities to live close to workplaces as well as have access to a high density of shops, services, and recreational facilities may be useful for reducing workers’ sedentary time. Future investigations with improvements in research design and measurements are needed to more deeply understand the impacts of workplace neighbourhood environments on workers’ physically-active and sedentary behaviours.

## Supplementary information

**Additional file 1:** **Supplementary Material 1.** Search terms and syntax for the literature search.

**Additional file 2:** **Supplementary Material 2.** Quality Assessment Tool for Observational Cohort and Cross-Sectional Studies.

## Data Availability

Not applicable.
