# Supplementary material for: Workplace neighbourhood built environment and workers’ physically-active and sedentary behaviour: a systematic review of observational studies
Source: Int J Behav Nutr Phys Act. 2020 Nov 20;17:148. doi: 10.1186/s12966-020-01055-x (PMC7678125; doi:10.1186/s12966-020-01055-x)
Supplement: Supplementary file 1 — Additional file 1: Supplementary Material 1. Search terms and syntax for the literature search. [file 12966_2020_1055_MOESM1_ESM.docx]

# Supplementary Material 1. Search terms and syntax for the literature search

| Set | Concept | Search Statement |
| --- | --- | --- |
| 1 | Physical activity | (physical activity OR walking).ti. |
| 2 | Sedentary behaviour | (sedentary behaviour OR prolonged sitting).ti. |
| 3 | Environmental variables | (workplace OR work site OR worksite OR office environment OR work space).ti. AND (neighbourhood OR walkability OR destination OR sidewalk OR pathway OR connectivity OR aesthetic OR safety).tx. |
| 4 | Combine sets | 1 OR 2 AND 3 |
| 5 | Limit | peer-reviewed journal articles.pt. |
| 6 | Limit | English.la. |
| 7 | Limit | January 2000-October 2019.dt. |
| 8 | Combine sets | Limit 4 to 5, 6, and 7 |
